# Microbial and Phenyl Acid Dynamics during the Start-up Phase of Anaerobic Straw Degradation in Meso- and Thermophilic Batch Reactors

**DOI:** 10.3390/microorganisms7120657

**Published:** 2019-12-05

**Authors:** Eva Maria Prem, Rudolf Markt, Nina Lackner, Paul Illmer, Andreas Otto Wagner

**Affiliations:** Department of Microbiology, Universität Innsbruck, A-6020 Innsbruck, Austria; rudolf.markt@uibk.ac.at (R.M.); nina.lackner@uibk.ac.at (N.L.); paul.illmer@uibk.ac.at (P.I.); andreas.wagner@uibk.ac.at (A.O.W.)

**Keywords:** phenylacetate, phenylpropionate, phenylbutyrate, anaerobic degradation, straw from grain, *next-generation* sequencing, metagenomic predictions

## Abstract

Aromatic compounds like phenyl acids derived from lignocellulose degradation have been suspected to negatively influence biogas production processes. However, results on this topic are still inconclusive. To study phenyl acid formation in batch reactors during the start-up phase of anaerobic degradation, different amounts of straw from grain were mixed with mesophilic and thermophilic sludge, respectively. Molecular biological parameters were assessed using *next-generation* sequencing and qPCR analyses. Metagenomic predictions were done via the program, *piphillin*. Methane production, concentrations of phenylacetate, phenylpropionate, phenylbutyrate, and volatile fatty acids were monitored chromatographically. *Methanosarcina* spp. was the dominant methanogen when high straw loads were effectively degraded, and thus confirmed its robustness towards overload conditions. Several microorganisms correlated negatively with phenyl acids; however, a negative effect, specifically on methanogens, could not be proven. A cascade-like increase/decrease from phenylacetate to phenylpropionate, and then to phenylbutyrate could be observed when methanogenesis was highly active. Due to these results, phenylacetate was shown to be an early sign for overload conditions, whereas an increase in phenylbutyrate possibly indicated a switch from degradation of easily available to more complex substrates. These dynamics during the start-up phase might be relevant for biogas plant operators using complex organic wastes for energy exploitation.

## 1. Introduction

In the next few decades, humankind will increasingly face challenges due to global energy demand and the resultant efforts to reduce greenhouse gas emissions from fossil fuel utilisation and combustion. In this regard, biogas production by anaerobic degradation of an organic material, along with hydroelectric power, wind energy, or solar power, represents a further step towards independence from non-renewable energy sources [1]. Locally gathered organic wastes are of special interest as their energy exploitation is considered economically effective and sustainable [2]. One drawback of using (pre-treated) organic waste products is the concurrent entry of undesirable compounds like ammonia [3] or aromatic compounds [4,5,6] that can cause severe disturbances during the cascade-like proceeding anaerobic degradation process [7]. Consequently, this can lead to restricted biogas production performances and thus to tremendous financial problems for the operators who are dependent upon specified quantities of methane to economically sustain the facility [5,8,9]. Moreover, reactor malfunction might lead to additional disposal costs because efficient treatment, and thus size reduction of the waste is not possible. Biogas plants are normally operated at mesophilic (25–40 °C) or thermophilic (>45 °C) temperatures. Due to the higher process robustness and lower energy input, mesophilic plants are far more abundant than thermophilic reactors. However, thermophilic plants have a clear advantage over mesophilic plants in terms of pathogen removal, exploitation efficiency, biochemical reaction rate, and occupation of ecological niches [10,11].

Aromatic compounds like benzoate [12], p-cresol [5], or phenylacetate (PAA) [13,14] are associated with process instability and disturbances [9]. One major source for aromatic compounds are lignocellulosic materials often found in municipal and agricultural waste materials [15]. Their common characteristic is a benzene ring, which is thermodynamically very stable [16]. The degradation of aromatic compounds, and particularly the cleavage of the benzene ring is possible under anaerobic conditions; however, the microorganisms involved depend on their ability to express specific enzymes [12,17,18]. Phenyl acids are mostly degraded to benzoyl-CoA which consequently enters the central benzoyl-CoA pathway [19,20,21] responsible for the actual cleavage of the benzene ring by the enzyme 6-oxocyclohex-1-ene-1-carbonyl-CoA hydrolase [16,18].

PAA, for example, was the focus of several studies concerning aromatic compound formation and their effects on anaerobic degradation [5,8,9,13,14]. However, information on the effects of PAA on the microbial community in general and on methanogenic *Archaea*, in particular, is still inconclusive due to the variety of aromatic compound sources, anaerobic digestion (AD) temperature regimes, and the inocula investigated. For instance, Hecht et al. (2009) [9] documented the impact of PAA on the anaerobic digestion process but could not find any influence of PAA on mesophilic methanogenic *Archaea*. They considered PAA to be an appropriate indicator of impending process failures because of its low limit of detection [9]. Others found that hydrolytic and methanogenic activities, especially acetoclastic methanogenesis, can be restricted when PAA is present [14]. In another study, overload conditions caused the formation of PAA and phenylpropionate (PPA) but did not generally restrict methanogenic performance [22]. PPA was also found in batch reactors fermenting straw material [4].

The phyla *Firmicutes* and *Proteobacteria* were thought to be involved in anaerobic aromatic compound degradation [4,23]. For instance, the delta-proteobacterium *Syntrophus acidotrophicus* was shown to degrade benzoate in syntrophic associations with methanogens in benzoate enrichment cultures [23,24]. *Sporomusa* spp., an acetogenic microorganism of the phylum *Firmicutes*, is able to grow on methoxylated aromatic compounds like syringate or vanillate [23].

In comparison to other aromatic compounds, PAA and PPA, did not receive much attention so far [22] even though these compounds are important intermediates during anaerobic degradation of benzenes derived from both proteinaceous, as well as lignocellulose-rich materials [4,22]. Profound information on the effects of phenylbutyrate (PBA) on anaerobic digestion is even missing completely. Therefore, a better understanding of the dynamics of PAA, PPA, and PBA in anaerobic, methanogenic systems is still pending. 

In this study, mesophilic and thermophilic reactors were set up with straw from grain in different overload conditions to (i) evaluate the overall digestion start-up phase (28 days) of straw degradation, (ii) initiate anaerobic phenyl acid formation during the start-up phase of anaerobic straw degradation, and (iii) link the formation and/or degradation of PAA, PPA, and PBA to specific taxa, metabolic pathways, and enzymes. 

## 2. Materials and Methods

The mesophilic inoculum was taken from a biowaste co-utilizing wastewater treatment plant in Zirl (Austria) [25] with a reactor capacity of 1350 m^3^, an operation temperature of 39 (±0.2) °C, pH of 7.4 (±0.21), and total solids content of 2.2 (±0.04) g 100 g^−1^ fresh weight. The thermophilic inoculum was derived from the outlet sampling port of a thermophilic anaerobic digestion plant in Roppen (Austria) where about 2.500 tons of green waste and 6.200 tons of biowaste are treated per year [26], with a total reactor capacity of 900 m^3^, an operation temperature of 53 (±0.3) °C, pH of 7.9 (±0.44), and a total solids content of 26.2 (±2.0) g 100 g^−1^ fresh weight. Additional information regarding digester conditions and characteristics can be looked up elsewhere [22]. Plastic bottles filled with sludge were tightly sealed and immediately brought to the laboratory. For liquid handling, the sludge was sieved and diluted as described previously [27,28]. The headspace was exchanged with a N_2_/CO_2_ (70:30)-gas mixture. The prepared samples were incubated at 37 °C and 52 °C for 15 days (mesophiles) and 20 days (thermophiles), respectively, until the sum of volatile fatty acids (VFA) was <200 mg kg^−1^. Subsequently, the samples were stored at 4 °C until further use.

Straw from grain (straw) was air-dried, but otherwise not chemically, physically, or biologically (pre)treated. The straw was cut into pieces 4–7 cm long. The C/N ratio of the straw (ratio: 56) was analysed with a TruSpec^®^ CHN analyser (Leco, Germany) according to the manufacturer’s protocol. The straw was filled into 120 mL serum flasks, functioning as batch reactors, in different carbon-load concentrations with 3 (defined as low carbon load, LCL), 34 (defined as medium carbon load, MCL), and 170 (defined as high carbon load, HCL) mmol carbon-C reactor^−1^, respectively. 

A basal anaerobic broth based on previous investigations [29] was prepared and modified as follows (per litre): 0.4 g NaCl, 0.4 g MgCl_2_ × 6 H_2_O, 0.68 g KH_2_PO_4_, 0.18 g NaOH, 0.05 g CaCl_2_ × 2 H_2_O, 0.4 g NH_4_Cl, 0.5 g L-cysteine, 10.0 g sodium carboxymethylcellulose (CMC), 0.5 g yeast extract, 2.0 g sodium acetate, 1.0 g sodium formiate, 1 mL vitamin solution [29], 1 mL trace element solution SL-10 (German Collection of Microorganisms and Cell Cultures GmbH (DSMZ), Braunschweig, Germany), 2 mL sodium sulfide solution (120 g L^−1^ Na_2_S), and 1 mL resazurine solution (1.15 g L^−1^ resazurine). After the pH was adjusted with 0.1 M sodium hydroxide to 7.5 ± 0.2, 48 mL of the medium was filled into the 120 mL serum flasks which had previously been filled with straw (as described above). A control containing the anaerobic broth but no straw was also included and equally treated thenceforward. The sealing and headspace gas exchange took place according to previous protocols [22]. The flasks were subsequently autoclaved and cooled down before further use. 

For each temperature regime, a volume of 12 mL diluted inoculum was injected into each reactor. Subsequently, the reactors were incubated at 37 °C and 52 °C, respectively, extending over an anaerobic incubation period of 28 days. All variations were prepared in triplicate. Samples were taken on day 2, 4, 7, 14, 21, and 28. Liquid samples for pH, VFA, phenyl acids, and C/N_liquid_ were processed immediately or frozen at −20 °C. The pH of the samples was measured with pH indicator strips 4.0–9.0 (Merck, Germany). 

For each temperature regime, a PCR-DGGE approach [30,31] was conducted with all variants of day 0 to check for the same microbial community structure at the beginning of the experiment. Moreover, control samples of day 0, as well as samples of day 14 and 28 were used for *next-generation* sequencing (NGS) analyses.

VFA, total carbon, total nitrogen (C/N_liquid_ ratio), as well as phenyl acid analyses were done according to previous studies [22,28,32]. The gas over-pressure was measured with a GHM Greisinger GDH 200 sensor and used to calculate biogas and methane production [NmL] as described previously [27].

Liquid samples (1 mL) from day 0, 14, and 28 were centrifuged at 20,000 g for 15 min and resuspended in 1 mL sterile ¼ Ringer solution. Subsequently, DNA extraction was done using the Soil Extract II Kit DNA (Macherey-Nagel). 700 µL of each sample were filled in bead-tubes and centrifuged at 11,000 g for 10 min. The supernatant was discarded and buffer SL-1 (700 µL) and the enhanced lysis buffer (50 µL) were added. Each further extraction step was done according to the manufacturer’s manual. The DNA was eluted in 50 µL elution buffer. DNA quantity and co-extraction of contaminants (absorbance ratio 260/280 and 260/230) was checked via the NanoDrop 2000c™ system. 

For the quantification of methanogenic *Archaea*, the *mlas*-f/*mcrA*-r primer pair [33,34] targeting the methyl coenzyme M reductase subunit A (*mcrA*) gene was used. Analyses were done on a Corbett Life Science (Qiagen, the Netherlands) Rotor-Gene Q system. The PCR procedure was conducted as follows: initial denaturation at 95 °C for 10 min, followed by 45 cycles of denaturation (95 °C for 30 sec), annealing (66 °C for 30 sec), and extension (72 °C for 30 sec). A PCR solution of 20 µL contained 9 µL PCR Mix (SensiFast™ SYBR No-Rox Kit (2×) (Bioline, UK), 380 nM of each primer, 1 mM MgCl_2_, 20% Betaine Enhancer Solution (5×) (VWR International, Germany), and PCR-grade water to reach a final volume of 18 µL, as well as a 2 µL template (5 ng DNA µL^−1^). An eight-point standard curve using gene copies of *Methanosarcina thermophila* and a melt-curve analysis were included in the approach.

The NGS library was prepared in-house. The small subunit (SSU) rRNA gene primers 515f and 806r [35], according to the Earth Microbiome Project [36], were used to target the V4 region. The first PCR step, including the 16S rRNA primers and the Illumina^®^ adapter sequences, was performed as follows: initial denaturation at 95 °C for 5 min, followed by 30 cycles of denaturation (95 °C for 45 s), annealing (57 °C for 45 s), and extension (72 °C for 90 s). A final extension step of 72 °C for 10 min was set at the end of the PCR process. A PCR solution of 25 µL contained 12 µL PCR Mix (VWR Red Taq DNA Polymerase Master Mix Kit (2×)), 250 nM of each primer-adapter combination, 20% Betaine Enhancer Solution (5×), PCR-grade water to reach final volume of 24 µL, as well as 1 µL DNA template (5 ng DNA µL^−1^). The quality of the PCR products was checked with a 1.5% agarose gel using the dye GelGreen^®^ Nucleic Acid Gel Stain (Biotium, Fremont, CA, USA). The PCR products of the first step were diluted 1:5 and used as a template for a second amplification to attach the Illumina^®^ barcodes (i5 and i7). The same PCR procedure as in the first PCR step was used, except that only seven cycles were applied and the annealing temperature was set to 56 °C. The PCR products were again checked with a 1.5% agarose gel. Subsequently, final PCR products were quantified fluorometrically, as described previously [37]. The PCR products (15 ng) of each sample were pooled and purified with a Hi Yield^®^ Gel/PCR DNA Fragment Extraction Kit (SLG^®^, Gauting, Germany) and eluted in 50 µL Tris-HCl buffer. The DNA quantity was again measured via QuantiFluor^®^ dsDNA Dye (Promega, Madison, WI, USA). Co-extraction of contaminants was checked via the NanoDrop 2000c™ system. The final ready-to-load sample pool showed a DNA concentration of 19 ng µL^−1^ (260/280 absorbance ratio: 1.88) and was subsequently sent to Microsynth AG in Switzerland where the sequencing was done according to the company’s protocols. 

In total, 27 mesophilic, 27 thermophilic, as well as nine MOCK samples were analysed. Raw sample reads were processed using the program mothur version 1.39.5 [38] and the MiSeq SOP (July 2019) [39]. A contig file was created with the paired-end reads (4,428,969 sequences in total, 70,301 ± 14,082 sequences sample^−1^). After quality filtering (approx. 24% of the sequences were discarded), unique sequences were aligned to the SILVA V132 database (Appendix A). After another quality check and pre-clustering, chimeric amplicons were removed applying the VSEARCH algorithm (VSEARCH v2.3.4.). Sequence classification was done with the k-nearest neighbor (knn) algorithm. Sequences were binned to phylotypes based on their taxonomy. For a better comparability of samples while simultaneously ensuring an adequate coverage of the species richness, rarefaction curves were generated, and samples were normalised to 22,800 reads per sample [40]. The *Mantel* test showed that the similarity matrices prior to and after rarefaction did not differ significantly from each other (*R* > 0.99, *p* < 0.01, *N* = 9999). Quality-filtered sequences were uploaded to GenBank^®^ via the submission tool, *BankIt* (Appendix B). Information on the MOCK communities can be looked up in Appendix C.

After quality filtering and subsampling to 22,800 reads per sample, a FASTA file containing only representative sequences and an operational taxonomic unit (OTU) table was generated via mothur (version 1.42.1). The files were uploaded to https://piphillin.secondgenome.com (September 2019). The tool *piphillin* used the nearest-neighbor algorithm to pair 16S rRNA gene sequences to genomes [41]. The Kyoto Encyclopedia of Genes and Genomes (KEGG) database [42] of October 2018 was applied. The identity cut-off was set at 97%. The analyses focused on general biochemical pathways and on pathways regarding anaerobic degradation/turnover of aromatic compounds: degradation of aromatic compounds (KEGG orthology ko01220), phenylpropanoid biosynthesis (KEGG orthology ko00940), benzoate degradation (KEGG orthology ko00362), and aminobenzoate degradation (KEGG orthology ko00627).

After rarefaction analyses, meso- and thermophilic data were analysed separately, using only OTUs with a total abundance of ≥35 for each temperature regime. In mothur, the *get.coremicrobiome* command was applied to gain information on the microorganisms being present in every variant of the respective temperature regime [38,39]. For characterising microorganisms important for explaining the variation between the C-load samples (class) of each temperature regime (biomarker discovery), the *LEfSe* command was applied [43]. For an interactive visualisation of relative sequence abundances of meso- and thermophilic samples, respectively, the tool KRONA was used [44]. The significance cut-off was set at *α* = 0.05 for all analyses. Significant genera were shown with the program STAMP 2.1.3 (Parks et al., 2014). For that purpose, White’s non-parametric *t*-test (two-sided) was used to distinguish between variants [45]. Confidence intervals were provided via percentile bootstrapping (1000 permutations). The false discovery rate was controlled with the *Benjamini-Hochberg* procedure (B-H adjustment) [46]. Via the program PAST^®^ 3 [47], *Spearman’s* rank correlation analyses (*Spearman* rs) were done for all samples of day 28 for each temperature regime: Genera with a standard deviation below 3 over all samples of day 28 of each temperature regime were excluded; phenyl acids were log (x+1), and the OTU data box-cox (x+1) transformed. The false discovery rate was controlled with the B-H adjustment in Microsoft^®^ Excel^®^. Moreover, the *Mantel* test (Gower Similarity Index) was applied in PAST^®^ 3. For *piphillin* and biochemical analyses, the *Mann–Whitney* U test (M-W, two-sided) and the *Friedman* ANOVA (time series) were applied, respectively (Statistica™ 13 (TIBCO^®^ Software Inc.)). Graphical presentations of correlation analyses and methanogenic properties were done with SigmaPlot™ 14 (Systat^®^ Software Inc.), of general microbial properties with STAMP 2.1.3, and of biochemical and *piphillin* analyses with Statistica™ 13.

## 3. Results

### 3.1. Mesophilic Communities

#### 3.1.1. Methane Production, Acetate, and Phenyl Acid Concentrations

**Figure 1:**
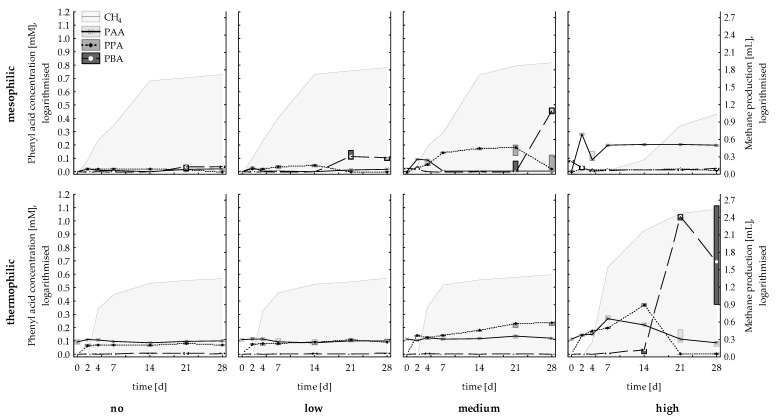
Cumulative methane production (grey area) and phenyl acid concentrations of no (controls), low, medium, and high C-load samples of the mesophilic (top) and thermophilic (bottom) biogas reactors, respectively, from day (d) 0 to day 28. For better comparability, results are log (x+1) transformed; marker points and boxes show means and percentiles (25–75%), respectively.

Methane production during the start-up phase of straw degradation is depicted in Figure 1 for the control, as well as the low (LCL), medium (MCL), and high (HCL) carbon load. The highest cumulative methane production was observed in MCL samples (85.3 ± 4.78 NmL CH_4_ cum), followed by LCL samples (70.0 ± 2.51 NmL CH_4_ cum), control samples (52.2 ± 2.71 NmL CH_4_ cum), and HCL samples (9.86 ± 1.44 NmL CH_4_ cum). After 28 days (start-up phase), approximately 24% (LCL), 13% (MCL), and 0.4% (HCL) of the theoretical maximum CH_4_ yield was achieved. The generation of phenyl acids under different C-load conditions is depicted in Figure 1. According to the *Friedman* ANOVA results, PAA, PPA, and PBA concentrations changed significantly in all variants during the incubation. The highest mean PAA concentration was detected in HCL samples on day 2 (123 ± 3 mg L^−1^ PAA), the highest mean PPA concentration in MCL samples on day 14 (73 ± 3 mg L^−1^ PPA), and the highest mean PBA concentration in MCL on day 28 (307 ± 24 mg L^−1^ PBA). For a comprehensive list of VFA concentrations, pH values, and C/N_liquid_ ratio, please refer to Table S1.

#### 3.1.2. Microbial Community Composition

**Figure 2:**
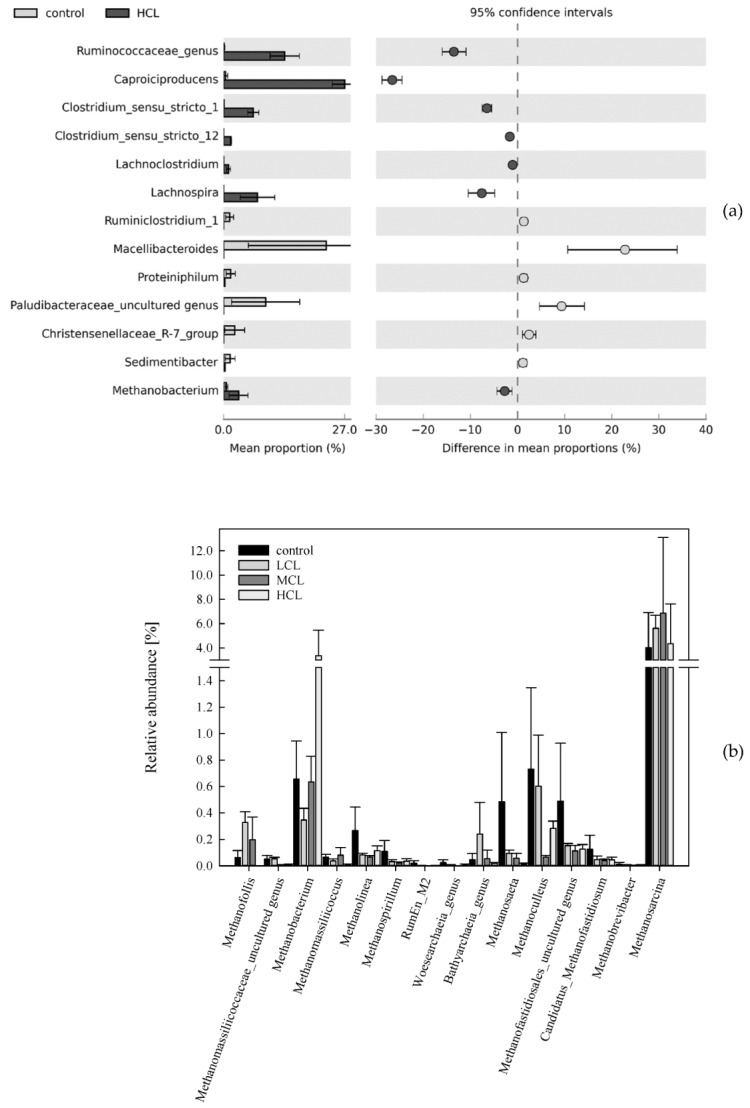
(**a**) Mean sequence proportions [%] of mesophilic phyla (*p* < 0.05, B-H adjusted, effect size > 1) of all control and high C-load (HCL) samples, (**b**) mean relative abundances [%] of methanogenic *Archaea* in all mesophilic controls and low (LCL), medium (MCL), and high C-load (HCL) samples.

After subsampling, 966 OTUs were detected for the mesophilic approach and 322 OTUs remained after removing OTUs with a total read abundance below 35. Over all the mesophilic samples, 32 phyla were detected, the most abundant were *Firmicutes*, *Bacteroidetes*, *Euryarchaeota*, *Chloroflexi*, and *Fibrobacteres*. Compared with the control samples, the abundance of genera like *Caproiciproducens*, *Ruminococcaceae* (unknown genus), and *Lachnospira* were significantly higher in HCL samples (Figure 2a). The relative sequence abundance of genera like *Bacteroides*, *Petrimonas*, or *Acetanaerobacterium* was significantly higher in MCL samples than in control samples; moreover, these taxa were also found in the respective core microbiome (Table 1). Furthermore, the first two were significant *LEfSe* biomarkers for MCL samples (Table 2). By contrast, *Macellibacteroides* spp. dominated in control samples on days 14 and 28, and its relative sequence abundance generally decreased with the straw load. For a comprehensive overview of the relative abundances in mesophilic samples, please refer to the KRONA file in the Supplementary Information (Figure S1).

When looking at correlations with phenyl acids specifically, *Ruminococcaceae* UCG-009 (genus level) was negatively (*p* < 0.05) correlated with PAA concentration (rs = −0.89). Information on other genera remained inconclusive.

The methanogenic community in the control, LCL, and MCL samples was dominated by *Methanosarcina* spp. (Figure 2b) which was a member of the core microbiome in those samples, whereas *Methanobacterium* spp. was listed in the core microbiome of HCL samples (Table 1). According to the *LEfSe* analysis, the latter was also a significant biomarker for HCL samples (Table 2). Via qPCR, the highest mean abundance of gene *mcrA* was observed for LCL samples of day 28 (4.86 × 10^7^ copies mL^−1^ sample), followed by MCL samples of day 14 (3.24 × 10^7^ copies mL^−1^ sample); the abundance was lowest in HCL samples of day 14 (2.73 × 10^6^ copies mL^−1^ sample). For a detailed list of *mcrA* abundance, please refer to Table S3.

### 3.2. Thermophilic Communities

#### 3.2.1. Methane Production, Acetate, and Phenyl Acid Concentrations

Methane production results are depicted in Figure 1. HCL samples showed the highest cumulative methane production of 350 ± 16.1 NmL CH_4_ cum reactor^−1^ (up to 21.3 NmL CH_4_ reactor^−1^ day^−1^). After 28 days (start-up phase), approximately 8% (LCL), 4% (MCL), and 15% (HCL) of the theoretical maximum CH_4_ yield (NmL) was achieved. The generation of phenyl acids under different C-load conditions is depicted in Figure 1. According to the *Friedman* ANOVA, the concentrations of all three phenyl acids varied significantly in LCL and HCL samples during the start-up phase. In MCL samples, only the PPA concentration changed significantly over time. No significant changes in PAA concentration could be observed in the controls. The highest phenyl acid concentrations were observed in HCL straw samples with a mean PAA concentration of 119 ± 9 mg L^−1^ PAA on day 7, a mean PPA concentration of 202 ± 8 mg L^−1^ PPA on day 14, and a mean PBA concentration of 1593 ± 80 mg L^−1^ PBA on day 21. For a comprehensive list of VFA concentrations, pH values, and the C/N_liquid_ ratio, please refer to Table S2.

#### 3.2.2. Microbial Community Composition

**Figure 3:**
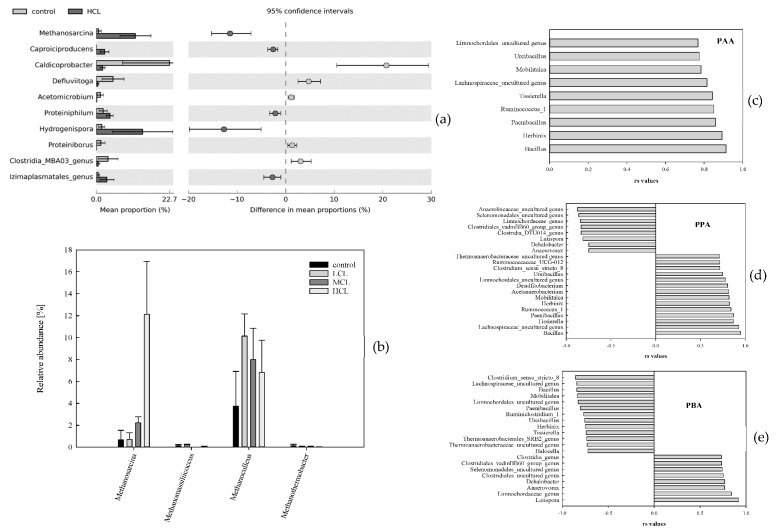
(**a**) Mean sequence proportions [%] of thermophilic phyla (*p* < 0.05, B-H adjusted, effect size > 1) of all control and high C-load (HCL) samples; (**b**) mean relative abundances [%] of methanogenic *Archaea* in all thermophilic control, low (LCL), medium (MCL), and high C-load (HCL) samples; and (**c**) significant *Spearman* correlations (B-H adjusted) between thermophilic genera and PAA, (**d**) PPA, and (**e**) PBA concentrations after 28 days.

After subsampling, 610 OTUs were detected for thermophilic samples. After OTU filtering, 139 OTUs remained for further analyses. Over all the thermophilic samples, 13 phyla were found; the most abundant were *Firmicutes*, *Euryarchaeota*, *Bacteroidetes*, *Thermotogae,* and *Tenericutes*. Compared with the control samples, the relative abundances of *Methanosarcina* spp. and *Hydrogenispora* spp. were significantly higher in HCL samples (Figure 3a). These two genera were also significant *LEfSe* biomarkers for the respective samples (Table 2). The genera *Tepidimicrobium*, *Proteiniphilum*, *Methanoculleus*, and *Ruminiclostridium* were part of every thermophilic core microbiome, irrespective of the C-load (Table 1). For a comprehensive overview of the relative abundances during incubation, please refer to the KRONA file in Figure S2.

For a detailed list of genera significantly correlating with PAA, PPA, and PBA on day 28, please refer to Figure 3c–e. The genera *Bacillus*, *Lachnospiraceae* (uncultured genus), *Tissierella*, *Paenibacillus*, *Ruminococcus* 1, *Herbinix*, *Mobilitalea*, *Limnochordales* (uncultured genus), and *Ureibacillus* positively (*p* < 0.05) correlated with PAA and PPA concentrations, respectively. Different genera positively (*p* < 0.05) correlated with the PBA concentration than with the PAA and PPA concentrations, respectively. For instance, genera such as *Lutispora* positively (*p* < 0.05) correlated with PBA, but negatively (*p* < 0.05) with the PPA concentration (Figure 3d,e).

*Methanosarcina* spp. was dominating the methanogenic community in MCL and HCL samples, and was thus part of the respective core microbiome (Table 1), whereas *Methanoculleus* spp. was the only methanogen in the core microbiome of the control and LCL samples. Moreover, *Methanoculleus* spp. and *Methanosarcina* spp. were also significant *LEfSe* biomarkers for the LCL and HCL samples, respectively (Table 2). Via qPCR, the mean *mcrA* gene copy number was highest in HCL samples with 2.93 × 10^8^ copies mL^−1^ sample on day 28 and 1.76 × 10^8^ copies mL^−1^ sample on day 14. For a detailed list of *mcrA* abundances, please refer to Table S3.

### 3.3. Prediction of Metagenomic Properties—Piphillin Analyses

**Figure 4:**
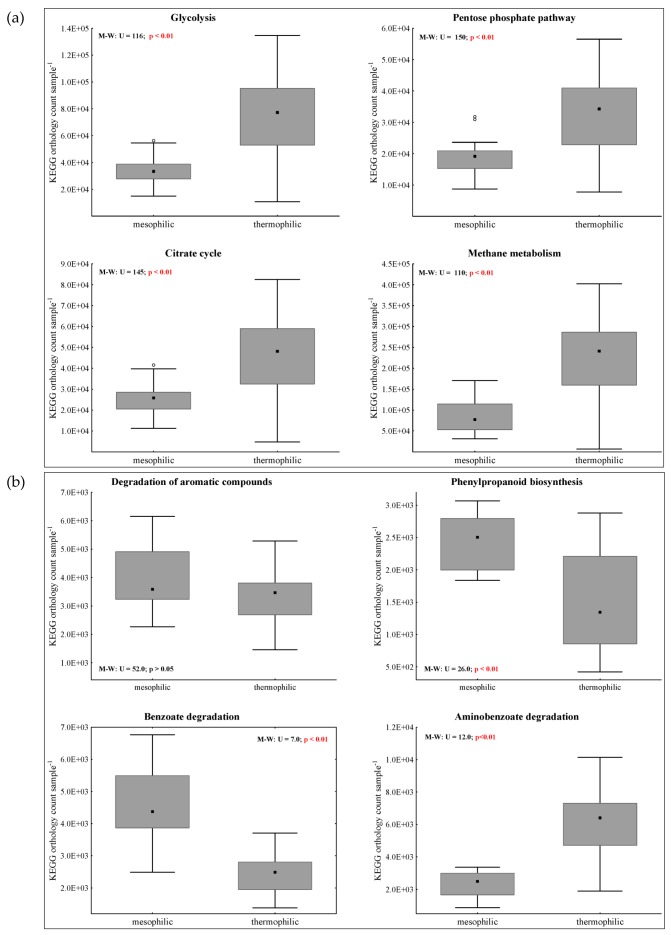
(**a**) The Kyoto Encyclopedia of Genes and Genomes (KEGG) orthology counts of general pathways of all meso- and thermophilic samples, respectively; (**b**) KEGG orthology counts of pathways relevant for anaerobic degradation of aromatic compounds of samples of day 28. The markers represent the median, the boxes show the upper to lower quartiles of each median, and the whiskers in the non-outlier range (coefficient 1) and circles represent outliers.

The analysis included 246 OTUs that exceeded the identity hit threshold of 97%. Furthermore, 281 genomes and 365 KEGG pathways were extracted for all mesophilic and all thermophilic samples. The orthology counts of classical biochemical pathways, such as glycolysis (ko00010), the pentose phosphate pathway (ko00030), citrate cycle (ko00020), and methane metabolism (ko00680) were significantly higher in thermophilic than in mesophilic samples (Figure 4a). Aminobenzoyl degradation (ko00627) was also more abundant in thermophilic samples; however, when looking specifically at pathways for benzene derivate turnover (ko00940 and ko00362), significantly more orthology counts could be observed for mesophilic samples (Figure 4b).

## 4. Discussion

The species richness was considerably higher in meso- than in thermophilic samples (after subsampling, 32 phyla were found for mesophilic and 13 phyla for thermophilic samples). Anaerobic mesophilic consortia are often considered quite resistant to disturbances due to functional/microbial redundancies [48]. However, a high species richness could also indicate that a stressed community tries to circumvent suboptimal conditions [49]. The applied mesophilic microbial community was derived from a wastewater treatment plant running at low carbon levels, with *Methanosaeta* spp. being the dominant methanogen [32]. Due to its high affinity for acetate, *Methanosaeta* spp. was previously shown to outcompete *Methanosarcina* spp. at low acetate concentrations (<1 mM acetate) [3]. These properties indicate that the native mesophilic microbial consortium was primarily adapted to low carbon loadings, and thus could not cope with a sudden availability of high carbon concentrations in the HCL samples. Consequently, severe disturbances in the form of a pH drop, an accumulation of VFAs (Table S1), and poor methane production performance (Figure 1) occurred. Moreover, the PAA concentration was highest in HCL samples, which supports previous assumptions that PAA could function as an early indicator for reactor disturbances/imbalances [9,13,22]. Most genera listed as significant *LEfSe* biomarkers (Table 2) and significant genera for mesophilic HCL samples are part of the order *Clostridiales* and depicted in Figure 2a. With a relative sequence abundance of 46%, *Ruminococcaceae* was the dominant family in HCL samples on day 28 (Figure S1). *Ruminococcaceae* (*Caproiciproducens* spp. and an unknown genus) and *Lachnospiraceae* (*Lachnospira* spp., Table 1) are known for their ability to persist on plant fibres and degrade complex plant compounds [50,51]. In mesophilic HCL samples, those fibrolytic microorganisms were probably responsible for an increase in H_2_ concentration at the beginning of the start-up phase (26.7 ± 2.65 NmL H_2_ on day 2) and for a high accumulation of VFAs at the end of the start-up phase (Table S1). Some representatives of the genus *Methanobacterium*, which are hydrogenotrophic methanogens and found in HCL reactors, are acidophilic and able to bear up against acetoclastic methanogens at low pH regimes [52]. However, even though *Methanobacterium* spp. was of importance for mesophilic HCL samples (Figure 2b, Table 1 and Table 2), methane production remained very low (Figure 1) in mesophilic reactors. 

During mesophilic incubation, methane production was highest in MCL samples. Despite the effective methane conversion, the anaerobic degradation process was still in the start-up phase, as only 13% of the theoretical maximum CH_4_ yield was achieved. The methanogenic process was dominated by the acetoclastic methanogen *Methanosarcina* spp. (Figure 2b), which was previously described as a heavy-duty methanogen, known for its robustness towards different impairments, including overload conditions [53,54]. However, this is quite controversial, as *Methanosarcina* spp. was also shown to be quite susceptible to process imbalances, like high ammonia concentrations, which in turn could lead to a shift towards syntrophic acetate oxidation (SAO)-induced hydrogenotrophic methanogenesis [3]. Due to the beneficial C/N_liquid_ ratios (Table S1) and the relatively high growth rates of *Methanosarcina* spp. [3], it can be assumed that *Methanosarcina* spp. had an advantage over other acetate consuming microorganisms, although acetate concentrations were high right from the beginning (>1 mmol L^−1^). However, despite the low abundances of hydrogenotrophic methanogens and syntroph acetate oxidising bacteria (Table 1, Figure 2b) and thus the minor role of SAO-induced hydrogenotrophic methanogenesis, it should be mentioned that the SAO pathway might still be active. The acetate was provided in the long-term by fermenters like *Bacteroides* spp., *Petrimonas* spp. [55], or *Acetanaerobacterium* spp. [56] (Table 1 and Table 2). At the end of the incubation period, a steep increase in PBA concentration could be observed in MCL samples (Figure 1) but did not adversely impact methane production. The increase in PBA concentrations might indicate a switch from easier towards more recalcitrant substrate utilisation by the microbial community. Several genera were associated with mesophilic MCL samples of day 28, and thus probably with mesophilic PBA formation on day 28. For instance, the relative sequence abundance of *Papillibacter* spp. was highest in MCL samples on day 28 (approx. 0.7%). *Papillibacter cinnamivorans* was previously shown to degrade cinnamate to acetate and benzoate [57] without degrading the phenyl ring itself. It can be hypothesised that the genus is also capable of degrading the aliphatic side chain of other aromatic compounds, leading to an increased PBA concentration [57]. However, this has to be evaluated in more detail in future studies. The relative abundance of *Ruminococcaceae* UCG-009 (genus level) was highest in MCL samples on day 28; furthermore, the genus was negatively correlated with PAA concentrations. This indicates that *Ruminococcaceae* were involved in the degradation of phenyl acids, as described previously [23]. Moreover, orthology counts for phenyl compound degradation were higher in mesophilic than in thermophilic samples (Figure 4b); this is probably due to the higher species richness and functional redundancies generally assumed for mesophilic digestion systems. However, anaerobic phenyl ring cleavage by the enzymes 6-oxocyclohex-1-ene-1-carbonyl-CoA hydrolase (K07539) could not be proven by the data of this study. Despite that, the genus *Syntrophus*, which was previously shown to degrade the benzene ring in syntrophic interactions [24], could also not be found for meso- and thermophilic samples. 

Despite the fact that the species richness of thermophilic reactors was considerably lower than that of mesophilic ones, the methane yield was higher in thermophilic than in the mesophilic samples after 28 days over all samples (Figure 1). Most strikingly, mesophilic samples showed the highest cumulative methane production at MCL conditions, whereas thermophilic samples showed the best methane production performance at HCL conditions. Besides, higher orthology counts for general metabolic pathways and aminobenzoate degradation were found in thermophilic samples (Figure 4). In HCL samples, the methanogenic process was dominated by *Methanosarcina* spp., which out-competed SAO-induced hydrogenotrophic methanogenesis. The latter was primarily performed by *Tepidanaerobacter* spp. and/or *Syntrophaceticus* spp. in syntrophic interaction with *Methanoculleus* spp. in LCL and MCL samples (Table 1, Figure 3b). The reasons for the high abundance of *Methanosarcina* spp. at high overload conditions are probably similar to those in mesophilic samples. *Ruminococcaceae* (*Caproiciproducens* spp. (Table 1 and Table 2), *Ruminiclostridium* (Table 1), and *Ruminococcaceae* UCG-010 (Table 2)) was relevant for degradation of complex plant compounds in thermophilic HCL samples. Furthermore, the genus *Hydrogenispora* was a significant *LEfSe* biomarker for thermophilic HCL samples (Table 2 and Figure 3a) and was probably co-responsible for a long-term supply of acetate. *H. ethanolica*, the only described species of this genus, was shown to be a mesophilic organism capable of fermenting a variety of carbohydrates. End products are mainly acetate, ethanol, and H_2_ [58]. There could also be thermophilic representatives of this genus involved in the hydrolytic, acidogenic, and acetogenic degradation steps. The relative sequence abundances of *Sporomusa* spp., which was shown to grow on methoxylated aromatic compounds [23], were quite low for thermophilic MCL (0.06%) and HCL samples (0.03%); the genus was even missing in all mesophilic and thermophilic control and LCL samples. This indicates that methoxylated aromatic compounds were not relevant and/or the incubation conditions were not appropriate for *Sporomusa* spp., at least in mesophilic samples. 

No direct negative effects of phenyl acids could be observed on methanogens, which is in accordance with previous investigations [22]. A successive, cascade-like increase/decrease of PAA, PPA, and PBA could be observed for HCL samples (Figure 1)—the highest PAA concentration could be shown on day 7, the highest PPA concentration on day 14, and the highest PBA concentration on day 21 (1593 ± 80 mg L^−1^ PBA). The quite early increase of PAA and PPA concentrations indicates that both phenyl acids were derived from rather easily degradable compounds. By contrast, PBA concentration increased during the later start-up phase and increased concurrently with the C/N_liquid_ ratio. This phenyl acid pattern was found for both mesophilic MCL (Table S1) and—even clearer—for thermophilic HCL reactors (Table S2). Therefore, and due to the low VFA results at the end of the incubation period, the increase in PBA concentration possibly indicated the end of the start-up phase and the beginning of a second degradation phase in which more complex straw materials were utilised. This still has to be validated for other (lignocellulosic) materials; however, the described PAA-PPA-PBA pattern might be helpful for biogas operators when using different organic materials or switching to lignocellulose-rich materials. 

In thermophilic samples, one of the highest correlations could be shown between PAA and PPA, respectively, and the genus *Bacillus*. On day 28, the relative abundance of *Bacillus* spp. was highest in MCL samples. *Bacillus* spp. was primarily shown to degrade aromatic compounds aerobically [59,60]; however, as many *Firmicutes* and fermenters are associated with the anaerobic degradation of aromatic compounds [23], it is plausible that *Bacillus* spp. took part in the phenyl acid turnover, at least in thermophilic samples. Many genera positively correlating with PAA and PPA, like *Bacillus* or *Tissierella*, were negatively correlated with PBA concentration (Figure 3) which further supports the assumption that PBA was an intermediate in the second degradation phase, utilising more complex plant compounds. Moreover, a highly positive correlation between PBA and *Lutispora* spp. was observed. Little is known about this genus so far; however, it was previously associated with hydrolysis processes in mesophilic aromatic wastewaters [61].

## 5. Conclusions

In this study, a cascade-like PAA-PPA-PBA pattern could be observed for mesophilic medium- and for thermophilic high-load samples. PAA was probably derived from easily available substrates and was an early indicator for overload conditions. An increase in PBA indicated the end of the start-up and the beginning of the degradation of more complex materials. *Methanosarcina* spp. dominated in those samples, and thus confirmed its essential role for stabilising overloaded reactors. Although the role of phenyl acids during anaerobic digestion processes remains to be further elucidated, these dynamics during the start-up phase might be relevant for monitoring the process stability and the start of degradation of more recalcitrant waste portions in biogas plants.

## Figures and Tables

**Table 1:** List of mesophilic and thermophilic genera defining the respective core microbiome for controls, low (LCL), medium (MCL), and high C-load (HCL) samples of day 14 and 28 (n = 6). Genera with (*) were part of every core microbiome of the respective temperature regime (n = 27). Methanogens are listed in bold.

C Load	Mesophilic Core Microbiome	Thermophilic Core Microbiome	
**control**	***Methanosarcina*** *Macellibacteroides* *Anaerolineaceae* (uncultured genus) * Paludibacteraceae (uncultured genus)	*Caldicoprobacter* *Hydrogenispora* *Ruminiclostridium* * Clostridia DTU014 genus ***Methanoculleus*** * Proteiniphilum * Clostridiales vadinBB60_group genus	*Tepidimicrobium* * Tepidanaerobacter *Clostridium* sensu stricto 1 *Defluviitoga* *Christensenellaceae* (uncultured genus) *Firmicutes* (uncultured genus) *Clostridia* MBA03 genus
LCL	***Methanosarcina*** *Macellibacteroides* *Clostridia* DTU014 genus *Anaerolineaceae* (uncultured genus) * Proteiniphilum *Lachnospiraceae* (uncultured genus) *Paludibacteraceae* (uncultured genus) *Ruminiclostridium* 1 *Bacteroides* *Christensenellaceae* R-7_group *Petrimonas*	*Caldicoprobacter* *Hydrogenispora* *Ruminiclostridium* * Clostridia DTU014 genus ***Methanoculleus*** * Proteiniphilum * Clostridiales vadinBB60_group genus	*Herbinix* *Tepidimicrobium* * Tepidanaerobacter *Defluviitoga* *Christensenellaceae* (uncultured genus) *Firmicutes* (uncultured genus) *Clostridia* MBA03 genus
MCL	***Methanosarcina*** *Macellibacteroides* *Caproiciproducens* *Anaerolineaceae* (uncultured genus) * Proteiniphilum *Bacteroides* *Petrimonas* *Acetanaerobacterium*	***Methanosarcina*** *Caldicoprobacter* *Caproiciproducens* *Hydrogenispora* *Ruminiclostridium* * Clostridia DTU014 genus ***Methanoculleus*** * Proteiniphilum * Clostridiales vadinBB60_group genus *Lachnospiraceae* (uncultured genus) *Herbinix* *Ruminiclostridium* 1	*Tepidimicrobium* * Tepidanaerobacter *Clostridium* sensu stricto 1 *Defluviitoga* *Christensenellaceae* (uncultured genus) *Firmicutes* (uncultured genus) *Ruminococcaceae* UCG-010 *Izimaplasmatales* genus *Clostridia* MBA03 genus *Oxobacter* *Anaerocolumna* *Limnochordales* (uncultured genus)
HCL	*Caproiciproducens* *Clostridia* DTU014 genus *Anaerolineaceae* (uncultured genus) * Clostridium sensu stricto 1 *Clostridia* D8A-2 genus *Ruminococcaceae* genus *Lachnospira* Candidatus *Caldatribacterium* ***Methanobacterium*** *Clostridium* sensu stricto 12	***Methanosarcina*** *Caproiciproducens* *Ruminiclostridium* *	***Methanoculleus*** * Proteiniphilum * Tepidimicrobium *

**Table 2:** Mesophilic and thermophilic genera considered significant *LEfSe* biomarkers for controls, low (LCL), medium (MCL), and high C-load (HCL) samples, with a linear discriminant analysis (LDA) score ≥ 4.0. Methanogens are listed in bold.

Temperature	C-load (Class)	Sample Size	*LEfSe* Biomarkers (LDA Score ≥ 4.0)
mesophilic	control	9	*Anaerolineaceae* (uncultured genus) *Clostridia* DTU014 genus *Bacteroidetes* vadinHA17 genus
	LCL	6	*Macellibacteroides* *Paludibacteraceae* (uncultured genus) *Ruminiclostridium* 1 *Christensenellaceae* R-7 group *Lachnospiraceae* (uncultured genus) *Proteiniphilum* *Ruminiclostridium* *Dysgonomonadaceae* (uncultured genus) *Mobilitalea*
	MCL	6	*Bacteroides* *Petrimonas*
	HCL	6	*Caproiciproducens* *Ruminococcaceae* genus *Lachnospira* *Clostridium* sensu stricto 1 ***Methanobacterium*** *Clostridia* D8A-2 genus
thermophilic	control	9	*Defluviitoga* *Syntrophaceticus* *Clostridia* MBA03 genus *Lactobacillus*
	LCL	6	*Caldicoprobacter* ***Methanoculleus***
	MCL	6	*Ruminiclostridium* *Lachnospiraceae* (uncultured genus) *Herbinix* *Izimaplasmatales* genus *Anaerocolumna*
	HCL	6	*Hydrogenispora* ***Methanosarcina*** *Ruminococcaceae* UCG-010 *Caproiciproducens* *Proteiniphilum*

## Supplementary Materials

The following are available online at https://www.mdpi.com/2076-2607/7/12/657/s1, Table S1: Volatile fatty acid (VFA: acetate, propionate, i-butyrate and butyrate) concentrations, C/N_liquid_ and pH of mesophilic control, LCL, MCL and HCL samples of day 0, 2, 4, 7, 14 and 28. Table S2: VFA (acetate, propionate, i-butyrate and butyrate) concentrations, C/N_liquid_ and pH of thermophilic control, LCL, MCL and HCL samples of day 0, 2, 4, 7, 14 and 28. Table S3: *mcrA* copies mL^−1^ of meso- and thermophilic control, LCL, MCL and HCL samples of day 0, 14 and 28. Figure S1: Interactive visualisation of mesophilic taxa of control, LCL, MCL and HCL samples of day 0, 14 and 28. Figure S2: Interactive visualisation of thermophilic taxa of control, LCL, MCL and HCL samples of day 0, 14 and 28.

## Appendix A

The SILVA V132 database [62] was downloaded from the SILVA homepage (https:/arb-silva.de/no_cache/download/archive/release_132/ARB_files/). Low-quality reads and chimera were removed with the program ARB [63]. The database was reduced to position 13k to 24k (according to *Escherichia coli* alignment coordinates in SILVA) via the program mothur [38]. Sequences containing more than six ambiguous bases were removed.

## Appendix B

BioProject ID: PRJNA565351 (mesophilic samples) and PRJNA565354 (thermophilic samples).

## Appendix C

Three different, defined MOCK communities were included to validate the NGS procedure. The ZymoBIOMICS™ Microbial Community standard (Zymo, containing eight bacterial and two yeast microorganisms, further referred to as Mock1) and the archaeon *Methanosarcina thermophila* DSM 1825 (DSMZ, German Collection of Microorganisms and Cell Cultures, further referred to as Mock2) were analysed separately, as well as in combination (50% genomic DNA Zymo, 50% genomic DNA *M. thermophila*, further referred to as Mock3). The MOCK communities were co-processed with reactor samples. The MOCK community was checked with RStudio^®^ using the packages *ggplot2* and *phyloseq* [64]. All bacterial and archaeal microorganisms (Mock1, Mock2, and Mock3) could be recovered at genus level; therefore, the validity and reliability of the applied strategies for DNA extraction, library preparation, and data processing were proven.
