# Supplementary material for: Microbial and Phenyl Acid Dynamics during the Start-up Phase of Anaerobic Straw Degradation in Meso- and Thermophilic Batch Reactors
Source: Microorganisms. 2019 Dec 5;7(12):657. doi: 10.3390/microorganisms7120657 (PMC6956005; doi:10.3390/microorganisms7120657)
Supplement: Supplementary file 1 [file microorganisms-07-00657-s001.pdf]

microorganisms

Supplementary

Microbial and phenyl acid dynamics during the start-up phase of anaerobic straw degradation in meso- and thermophilic batch reactors

Eva Maria Prem [1,*], Rudolf Markt [1], Nina Lackner [1], Paul Illmer [1] and Andreas Otto Wagner [1]

[1] Department of Microbiology, Universität Innsbruck, Innsbruck, Austria;
* Correspondence: eva.prem@uibk.ac.at;

Received: date; Accepted: date; Published: date

Table 1. Volatile fatty acid (VFA: acetate, propionate, i-butyrate and butyrate) concentrations, C/N_{liquid} and pH of mesophilic control, LCL, MCL and HCL samples of day 0, 2, 4, 7, 14 and 28. VFA concentrations are shown in mmol L^{-1}; mean values are presented with the respective standard deviations (SD).

Day	CL	Acetate		Propionate		i-Butyrate		Butyrate		C/N_{liquid}	pH	
		mean	SD	mean	SD	mean	SD	mean	SD		mean	SD
0	control	21.3	0.32	0.29	0.04	0.00	0.00	0.00	0.00	16	7.0	0.0
	LCL	22.6	1.58	0.40	0.02	0.00	0.00	0.00	0.00	16	7.0	0.0
	MCL	35.0	2.10	0.45	0.02	0.00	0.00	0.00	0.00	16	7.0	0.0
	HCL	77.5	8.50	0.38	0.04	2.21	0.27	0.00	0.00	17	6.9	0.1
2	control	21.1	0.34	0.62	0.14	0.00	0.00	0.46	0.31	16	7.5	0.0
	LCL	21.3	0.52	0.53	0.15	0.00	0.00	0.65	0.10	16	8.0	0.0
	MCL	25.3	0.33	1.34	0.19	0.00	0.00	2.70	0.96	18	7.2	0.1
	HCL	40.7	0.84	1.52	1.40	0.00	0.00	29.06	2.54	23	5.8	0.0
4	control	22.6	1.34	1.41	0.36	0.00	0.00	0.37	0.25	16	8.0	0.0
	LCL	25.0	1.44	2.06	0.05	0.00	0.00	1.10	0.10	16	7.8	0.0
	MCL	37.1	1.29	3.81	0.25	0.00	0.00	6.69	0.46	19	6.0	0.0
	HCL	59.9	4.72	3.06	0.45	0.00	0.00	41.49	1.04	28	5.0	0.0
7	control	25.0	1.01	2.30	0.16	0.00	0.00	0.72	0.17	15	7.6	0.1
	LCL	28.4	0.06	3.38	0.39	0.00	0.00	1.38	0.28	16	7.0	0.0
	MCL	43.4	3.26	4.12	0.25	0.00	0.00	7.18	0.70	19	5.5	0.0
	HCL	73.6	1.42	1.78	0.17	0.00	0.00	46.38	1.55	30	5.0	0.0
14	control	0.33	0.30	3.92	0.14	0.00	0.00	0.00	0.00	13	8.0	0.0
	LCL	0.38	0.08	6.36	0.44	0.00	0.00	0.00	0.00	14	7.8	0.0
	MCL	2.27	1.62	6.31	0.22	0.00	0.00	6.98	0.63	17	7.8	0.0
	HCL	82.6	3.16	3.05	0.82	0.00	0.00	48.67	0.64	31	4.8	0.0
21	control	0.10	0.17	3.86	0.14	0.00	0.00	0.00	0.00	13	7.8	0.0
	LCL	0.37	0.32	3.51	1.74	0.00	0.00	0.00	0.00	13	7.5	0.0
	MCL	0.76	1.32	7.62	1.02	0.00	0.00	0.00	0.00	17	7.5	0.0
	HCL	84.0	3.25	4.66	1.37	0.00	0.00	55.64	1.85	31	4.8	0.0
28	control	0.00	0.00	0.00	0.00	0.00	0.00	0.00	0.00	13	8.0	0.0
	LCL	0.00	0.00	0.00	0.00	0.00	0.00	0.00	0.00	13	8.0	0.0
	MCL	3.05	1.49	8.30	1.08	0.00	0.00	0.00	0.00	20	7.5	0.0
	HCL	84.0	1.43	5.49	0.89	0.24	0.01	53.01	3.38	30	4.5	0.0

Table 2. VFA (acetate, propionate, i-butyrate and butyrate) concentrations, C/N_{liquid} and pH of thermophilic control, LCL, MCL and HCL samples of day 0, 2, 4, 7, 14 and 28. VFA concentrations are shown in mmol L^{-1}; mean values are presented with the respective standard deviations (SD).

Day	CL	Acetate		Propionic acid		i-Butyric acid		Butyric acid		C/N_{liquid}	pH	
		mean	SD	mean	SD	mean	SD	mean	SD		mean	SD
0	control	17.7	3.40	0.08	0.08	0.24	0.10	0.03	0.05	12	7.5	0.0
	LCL	20.8	0.55	0.25	0.05	0.41	0.08	0.22	0.01	12	7.5	0.0
	MCL	24.6	0.42	0.23	0.13	0.64	0.03	0.59	0.09	12	7.4	0.1
	HCL	78.0	2.21	2.77	0.59	2.36	0.05	2.87	0.08	13	7.5	0.0
2	control	26.3	0.26	0.99	0.01	0.21	0.09	0.72	0.11	11	7.0	0.0
	LCL	27.3	0.25	1.09	0.16	0.21	0.10	0.66	0.15	11	7.0	0.0
	MCL	31.8	0.17	2.14	0.26	0.00	0.00	1.88	0.23	13	7.0	0.0
	HCL	45.9	0.43	1.84	0.07	0.12	0.01	6.42	0.04	13	6.8	0.0
4	control	29.1	0.71	0.88	0.02	0.10	0.01	1.54	0.09	12	7.3	0.0
	LCL	31.2	0.47	1.18	0.11	0.17	0.01	1.51	0.15	12	7.0	0.0
	MCL	35.6	0.53	2.30	0.04	0.19	0.01	3.52	0.31	13	7.0	0.0
	HCL	53.1	0.35	2.60	0.08	0.28	0.30	8.49	0.17	14	6.7	0.3
7	control	38.0	0.94	1.11	0.15	0.57	0.36	2.65	0.39	12	6.8	0.1
	LCL	41.7	0.25	1.32	0.13	0.72	0.13	2.14	0.46	12	6.7	0.1
	MCL	49.1	0.75	2.71	0.08	1.47	0.07	6.11	0.29	13	6.5	0.0
	HCL	68.7	3.42	5.51	0.28	0.36	0.20	13.35	0.49	15	6.0	0.0
14	control	46.1	1.12	3.03	0.30	0.98	0.16	2.13	0.10	11	6.5	0.0
	LCL	46.9	1.22	2.77	0.70	1.07	0.02	2.00	0.33	11	6.3	0.3
	MCL	52.7	0.99	3.18	0.14	1.82	0.65	6.77	0.53	12	6.0	0.0
	HCL	5.89	0.90	6.66	0.12	0.00	0.00	0.34	0.26	14	7.5	0.0
21	control	48.5	1.25	4.14	0.38	0.72	0.05	2.35	0.17	12	6.4	0.1
	LCL	49.3	1.38	2.30	0.79	0.84	0.08	2.17	0.37	12	6.3	0.1
	MCL	53.4	0.82	2.98	0.08	1.57	0.37	6.41	0.38	13	6.0	0.0
	HCL	2.89	0.60	2.03	0.60	0.00	0.00	0.97	1.34	31	7.5	0.0
28	control	51.3	0.48	3.18	0.38	1.08	0.12	2.79	0.11	10	6.5	0.0
	LCL	48.6	1.89	3.53	0.36	1.04	0.11	2.46	0.81	10	6.3	0.1
	MCL	53.2	1.77	3.13	0.24	1.13	0.56	6.33	0.37	11	6.0	0.0
	HCL	1.72	0.55	0.89	0.18	0.00	0.00	0.07	0.06	29	7.5	0.0

Table S3. *mcrA* copies mL^{-1} of meso- and thermophilic control, LCL, MCL and HCL samples of day 0, 14 and 28.

Temperature	Day	CL variant	mean	SD
mesophilic	0	control	3.44E+06	4.83E+04
	14	control	2.60E+07	4.12E+06
		LCL	3.10E+07	6.82E+06
		MCL	3.24E+07	8.22E+06
		HCL	2.73E+06	1.06E+06
	28	control	2.39E+07	1.43E+06
		LCL	4.86E+07	1.66E+07
		MCL	2.38E+07	9.62E+06
		HCL	5.93E+06	2.59E+06
thermophilic	0	control	1.57E+03	4.25E+02
	14	control	2.76E+05	1.33E+05
		LCL	4.35E+05	6.56E+04
		MCL	3.90E+05	1.69E+05
		HCL	3.53E+06	6.76E+05
	28	control	1.45E+05	6.36E+04
		LCL	1.92E+05	1.61E+05
		MCL	1.89E+05	7.73E+04
		HCL	5.86E+06	1.24E+05

Krona_straw_mesophilic.html

Figure S1. Interactive visualisation of mesophilic taxa of control, LCL, MCL and HCLsamples of day 0, 14 and 28.

Krona_straw_thermophilic.html

Figure S2. Interactive visualisation of thermophilic taxa of control, LCL, MCL and HCL samples of day 0, 14 and 28.

© 2019 by the authors. Submitted for possible open access publication under the terms and conditions of the Creative Commons Attribution (CC BY) license (http://creativecommons.org/licenses/by/4.0/).
